# Genome-Wide Screening of Salt Tolerant Genes by Activation-Tagging Using Dedifferentiated Calli of *Arabidopsis* and Its Application to Finding Gene for *Myo*-Inositol-1-P-Synthase

**DOI:** 10.1371/journal.pone.0115502

**Published:** 2015-05-15

**Authors:** Aftab Ahmad, Yasuo Niwa, Shingo Goto, Kyoko Kobayashi, Masanori Shimizu, Sohei Ito, Yumiko Usui, Tsutomu Nakayama, Hirokazu Kobayashi

**Affiliations:** 1 Laboratory of Plant Molecular Improvement, Graduate School of Nutritional and Environmental Sciences, University of Shizuoka, 52–1 Yada, Suruga, Shizuoka 422–8526, Japan; 2 Laboratory of Protein Engineering, Graduate School of Nutritional and Environmental Sciences, University of Shizuoka, 52–1 Yada, Suruga, Shizuoka 422–8526, Japan; 3 Laboratory of Molecular Fooineering, Graduate School of Nutritional and Environmental Sciences, University of Shizuoka, 52–1 Yada, Suruga, Shizuoka 422–8526, Japan; University of Western Sydney, AUSTRALIA

## Abstract

Salinity represents a major abiotic stress factor that can adversely limit the production, quality and geographical distribution of crops. In this study we focused on dedifferentiated calli with fundamental cell functions, the salt tolerance of which had not been previously examined. The experimental approach was based on activation tagging without regeneration of plants for the identification of salt-tolerant mutants of *Arabidopsis*. Among 62,000 transformed calli that were screened, 18 potential mutants resistant to 150 mM NaCl were obtained. Thermal asymmetric interlaced (TAIL)-PCR was performed to determine the location of T-DNA integration in the genome. In one line, referred to as *salt tolerant callus 1* (*stc1*), expression of a gene [At4g39800: *myo*-inositol-1-P-synthase 1 (MIPS1)] was considerably enhanced in calli. Plants regenerated from calli showed tolerance to salt in germination and subsequent growth. Retransformation of wild-type *Arabidopsis* with *MIPS1* conferred salt tolerance, indicating that *MIPS1* is the causal gene. The over-expression of *MIPS1* increased the content of total inositol. The involvement of *MIPS1* in salt tolerance through the fundamental cell growth has been proved in *Arabidopsis*.

## Introduction

Salinity represents a major abiotic stress factor that imposes serious threats to agricultural industries worldwide. Immoderate irrigation by underground water has resulted in the accumulation of salt in affected regions that collectively span an area estimated to be equivalent to the total area of the United States (ca. 1,000 km^2^). Salinity imposes both hyperionic and hyperosmotic stress that can disrupt homeostasis both at cellular and whole-plant levels. Altered osmotic potential results in molecular damage that ultimately hinders growth and can even lead to death [1, 2]. Some of the most severe effects resulting from salinity include the disruption of cell membranes, generation of reactive oxygen species, reduction in enzymatic and photosynthetic activities, and decreased nutrient acquisition [3]. Therefore, an understanding of plant salt regulatory systems and the engineering of salt tolerance are fundamental and critical areas that require attention [4].

Determining the molecular mechanisms of salt tolerance is critical for efforts aimed at the successful breeding or genetic engineering of salt-tolerant crops [5]. Most studies involving either loss of function, gain of function, or microarrays for the identification of components related to salt regulatory mechanisms have been performed using whole plants [6–10]. Whole-plant investigations of salt tolerance are thorough, and have revealed a variety of complex structures and tissue-specific processes. Moreover, as roots are in direct contact with salt, almost all studies have focused on shoot parts. Recently, the tissue-specific roles of salt-overly sensitive 3 (SOS3) and SOS3-like calcium binding protein 8 (SCABP8) have been reported for salt tolerance in roots and shoots, respectively [11], indicating that tissue- and organ-specific mechanisms of salt tolerance must be considered. In contrast, cultured cells provide an alternative model for tissue-specific studies with all the basic functions of plant cells, and overcome the complexity of whole plants.

Mutations involving loss of functions have been used successfully to identify the functions of genes, and mutation strategies involving gain of functions have been developed to compensate for genetic redundancy. While a number of technologies have been developed to obtain gain-of-functions mutations, activation tagging through quadruple enhancers [12] is a procedure that has been used effectively for this purpose. A successful example of the application of activation tagging concerns discovery of the roles of the cytokinin signal transduction gene (*CKI1*) [13]. In an effort to determine the mechanisms underlying salt tolerance in cells possessing basal survival functions, we have initiated the screening and characterization of *Arabidopsis* mutants tolerant to high NaCl concentrations in dedifferentiated cells utilizing a methodology based on activation tagging. In one of the mutants, a gene for *myo*-inositol-1-P-synthase (MIPS1), which catalyzes the first step of inositol biosynthesis from glucose-6-P, confirmed the salt tolerance both at cellular and plant levels.

## Materials and Methods

### Transformation and Selection of Mutants

Wild-type *Arabidopsis thaliana* (ecotype Col-0) and its progeny were used for the generation of activation-tagged mutant lines. Unless otherwise indicated, seeds were surface-sterilized, vernalized at 4°C for one week, and then sown on solidified Murashige-Skoog (MS) medium containing 0.2% Gellan gum (San-Ei Gen F.F.I., Inc., Toyonaka, Japan) [9]. Following 7 days of incubation in growth chambers (20°C, continuous fluorescent light), 15 to 25 seedlings were transferred into flasks containing liquid MS and subsequently grown with shaking at 80 rpm for 2 weeks. Cultured roots were then detached from green tissues (stem and leaves) and cut into small pieces (3 to 6 mm). These pieces were then transferred into callus-inducing medium (CIM) (MS supplemented with 0.5 μg/mL 2,4-D and 50 ng/mL kinetin) [14] and incubated in growth chambers for 5 days.

The roots were infected with *Agrobacterium tumefaciens* GV3101 harboring pRi35ADEn4, a binary vector for activation tagging [15]. Following 1 week of co-culturing, the roots were washed with liquid CIM supplemented with 0.1 mg/mL cefotaxime (Sanofi Aventis, Tokyo). The roots were then incubated on CIM in the presence of 0.2 mg/mL vancomycin (Merck, Osaka, Japan) and 0.1 mg/mL cefotaxime to inhibit the proliferation of *A*. *tumefaciens*, in addition to 0.1 μg/mL chlorsulfuron (Dr. Ehrenstorfer GmbH, Augsburg, Germany) for transformant selection over a period of 3 weeks. Finally, transformed calli were then transferred to CIM supplemented with 0.2 mg/mL vancomycin, 0.1 mg/mL cefotaxime, 0.1 μg/mL chlorsulfuron and 150 mM NaCl. Mutants were repeatedly selected on the medium. Following an initial selection at 150 mM NaCl, secondary selection was performed at 200 mM to 250 mM NaCl.

### Verification of T-DNA Inserts by PCR

Genomic DNA was isolated from calli that grew on CIM containing 0.1 μg/mL chlorsulfuron using Isoplant (NipponGene, Toyama, Japan) or as previously described [16]. The DNA was then subjected to PCR analysis to amplify an approx. 200-bp fragment of P*35S*-*ALS-SU* ^r^ using primers 35SminiL-fd [17] and ALS22-rv [15]. The PCR products were subjected to agarose gel electrophoresis using 3% (w/v) Agarose 21 (NipponGene, Toyama, Japan).

### Determination of Insertion Location on Chromosomes by TAIL-PCR

Genomic DNA was isolated from mutants and TAIL-PCR [18, 19] was performed using AD and T-DNA end primers [15, 19]. Purified fragments following tertiary PCR were sequenced directly and the flanking sequences obtained were subjected to a BLAST search using the *Arabidopsis* Information Resource (TAIR, http//:www.arabidopsis.org). Finally, specific primers were designed and used in combination with T-DNA-specific primers to amplify specific fragments which were subsequently sequenced to confirm the insertion sites.

### Real-Time PCR Analysis

Total cellular RNA was extracted using Isogen (NipponGene) and treated with RNase-free DNase (Takara, Otsu, Japan). The RNA was then subjected to cDNA synthesis using the First Strand cDNA Synthesis Kit (Roche, Indianapolis) and real-time PCR was conducted using the LightCycler Quick System 330 (Roche). For each reaction, 2 μL of diluted cDNA (equivalent to 200 pg of total cellular RNA) was mixed with 10 μL of SYBR green PCR master mix (Takara) and 10 pmol each of the forward and reverse primers in a final volume of 20 μL. PCR conditions comprised 45 cycles at 95°C for 5 s and 60°C for 20 s. The amplification was followed by a thermal denaturation step to generate dissociation curves which verified the amplification specificity. As an internal standard, the actin 2 gene *ACT2* [20] was used for the normalization of transcript levels.

### Regeneration of Plants

Calli grown on CIM supplemented with 150 mM NaCl were transferred to shoot-inducing medium (SIM) supplemented with 0.15% Gellan gum, 3-indolacetic acid (IAA) (final concentration, 0.15 μg/mL), N6-(2-isopentenyl) adenine (2-iP) (final concentration, 5μg/mL) and 0.1 μg/mL chlorsulfuron. Following the emergence of shoots (2 to 3 weeks), calli were separated from the shoots and both calli and shoots were transferred to fresh SIM. When shoots had reached a length of ca. 4 to 10 mm, calli were carefully removed with fine-pointed forceps and the shoots were transferred to root-inducing medium (RIM) supplemented with IAA (0.5 mg/mL) and 0.15% agar. Roots developed after ca. 3 weeks. When the roots were several millimeters long, plantlets were removed and roots were gently rinsed with water to remove any residual Gellan gum. Plantlets were transferred to soil and pots were covered with Saran Wrap to maintain a high humidity. Seeds were collected after 1 month and germinated on CIM supplemented with 0.1 μg/mL chlorsulfuron. Finally, PCR was performed to confirm the presence of the transgene.

### Salt Stress Treatment of Calli

Approx. 62,000 calli were selected on chlorsulfuron and maintained by subculturing at 3-week intervals over a period of 3 to 4 months. For the stress treatment of calli, wild-type and *stc1* calli were cultured on CIM supplemented with 0.1 μg/mL chlorsulfuron and either 150 mM or 200 mM NaCl.

### Salt Stress Treatment of Regenerated Plants

T2 and T3 homozygous and wild-type plants were screened for salt tolerance. Seeds were surface-sterilized and rinsed 5 times with sterile water. Following rinsing, seeds were resuspended in 0.2% agar and kept at 4°C in the dark for 7 d before being transferred to MS plates supplemented with 100 mM or 150 mM NaCl. After 1 month, plants that had survived and continued to grow were counted and analyzed.

### Construction for over-expression (OX) of *MIPS1* by Retransformation

The *MIPS1* open reading frame (ORF) was amplified using primers 5’-AGAAGCATCACTAGTTCACATGCA-3’ (forward) and 5’-CAAACCTCGAGACAAATTAAAGA-3’ (reverse). The ORF was first cloned into pBlueScript (Fermnetas, USA). Following sequencing, the *MIPS1* ORF was cloned under the control of the CaMV 35S promoter with 4 copies of its enhancer in the binary vector pBCH1 [21] to generate pBCH1-EN-MIPS. *A*. *tumefaciens* GV3101 was then transformed using this construct by means of electroporation.

### Generation of Retransformed Plants and Calli

The *MIPS1* was introduced under the control of CaMV 35S promoters both into plants and calli to investigate its role in salt tolerance at plant and cellular levels. In an effort to investigate the functions of MIPS1 in plants, 4- or 5-week-old wild-type plants were transformed using the floral-dip method [22] and T0 seeds were collected and planted on MS containing hygromycin. Twenty-four T0 plants resistant to hygromycin were transferred to soil so that T1 seeds could be collected. The T1 seeds of each plant were screened on hygromycin plates to determine the ratio of resistant and sensitive seedlings. Among the 24 plants screened, 4 plants were found to contain a single copy of T-DNA. Ten plants from these T1 seeds were transferred to soil so that T2 seeds could be collected. About 500 seeds of each plant were screened on hygromycin and finally 2 plants were found to be homozygous for T-DNA. Homozygous plants were transferred to soil so that T3 seeds could be collected. Roots of wild-type plants were infected with pBCH1-EN-MIPS and transgenic calli were generated on hygromycin-supplemented plates. Uninfected roots were unable to produce calli on hygromycin plates, but were able to grow on CIM.

### Salt Stress Treatment in Retransformed Plants and Calli

Plant seeds were surface-sterilized and placed in the dark for 7 d. Finally, seeds were planted on MS containing different levels of NaCl. Specimen photographs were taken 2 to 3 weeks following transfer to stress medium. Calli induced from wild-type roots and transgenic plants, as well as calli directly transformed with *MIPS1*, were transferred to CIM supplemented with 150 mM NaCl and kept for 3 weeks. Growing calli were then transferred to new plates.

### Measurement of *Myo*-Inositol Contents

*Myo*-inositol contents were determined by the method described in Smart and Flores [23].

### Analysis of Protein Structures

Amino acid sequences of MIPSs were aligned using the program ClustalW [24].

## Results

### Mutants Obtained by Activation Tagging

**Fig 1:**
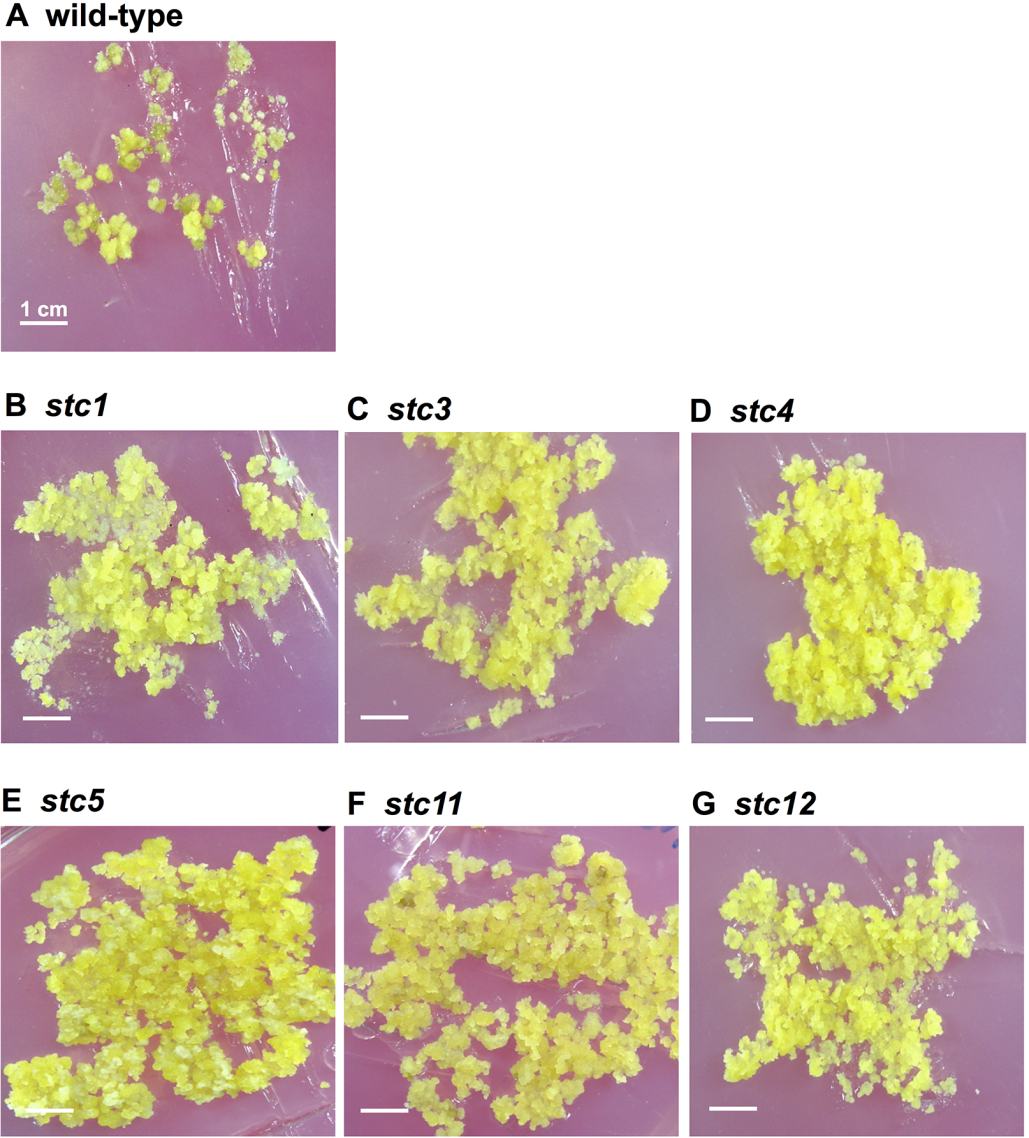
Phenotypes of calli of wild-type and *stc* mutants following NaCl stress. Yellow calli represent those that are growing, while the growth of those that are black or white has been severely affected by NaCl: (A) wild-type calli on 150 mM NaCl medium; (B) *stc1* calli on 150 mM NaCl medium; and (C, D, E, F and G) *stc3*, *stc4*, *stc5*, *stc11* and *stc12* on 150 mM NaCl medium, respectively.

In order to find the key genes involved in salt tolerance at cellular levels of *Arabidopsis*, mutant calli were generated and characterized at a 150 mM NaCl-stress level. In an effort to investigate salt tolerance at the cellular level, roots of *Arabidopsis* were infected with *Agrobacterium tumefaciens* harboring the binary vector pRi35ADEn4 [15], which contained four copies of a 339-bp long cauliflower mosaic virus (CaMV) 35S enhancer [12] at the right border of the T-DNA and a gene for acetolactate synthase as a selectable marker [25]. Transformants were selected on callus-inducing medium (CIM) supplemented with chlorsulfuron (a primary compound of sulfonylurea) and subsequently screened on 150 mM NaCl-containing medium (Fig 1). About 62,000 activation-tagged calli were screened and 40 mutants were selected with an initial screening at 150 mM NaCl. For the selection of mutant calli, the transfer of calli was performed four times at 3-week intervals between each transfer. In the second screening, the 40 mutant calli selected initially were further screened on 200 mM NaCl medium. Eighteen mutants were finally selected for analysis. It was observed that the survival and proliferation rates of mutant calli were significantly higher at different NaCl stress levels compared with untransformed calli (Fig 1), while they grew similarly to the wild-type without salt stress as described below together with phenotypes of a *MIPS1*-knockout (KO) line.

**Fig 2:**
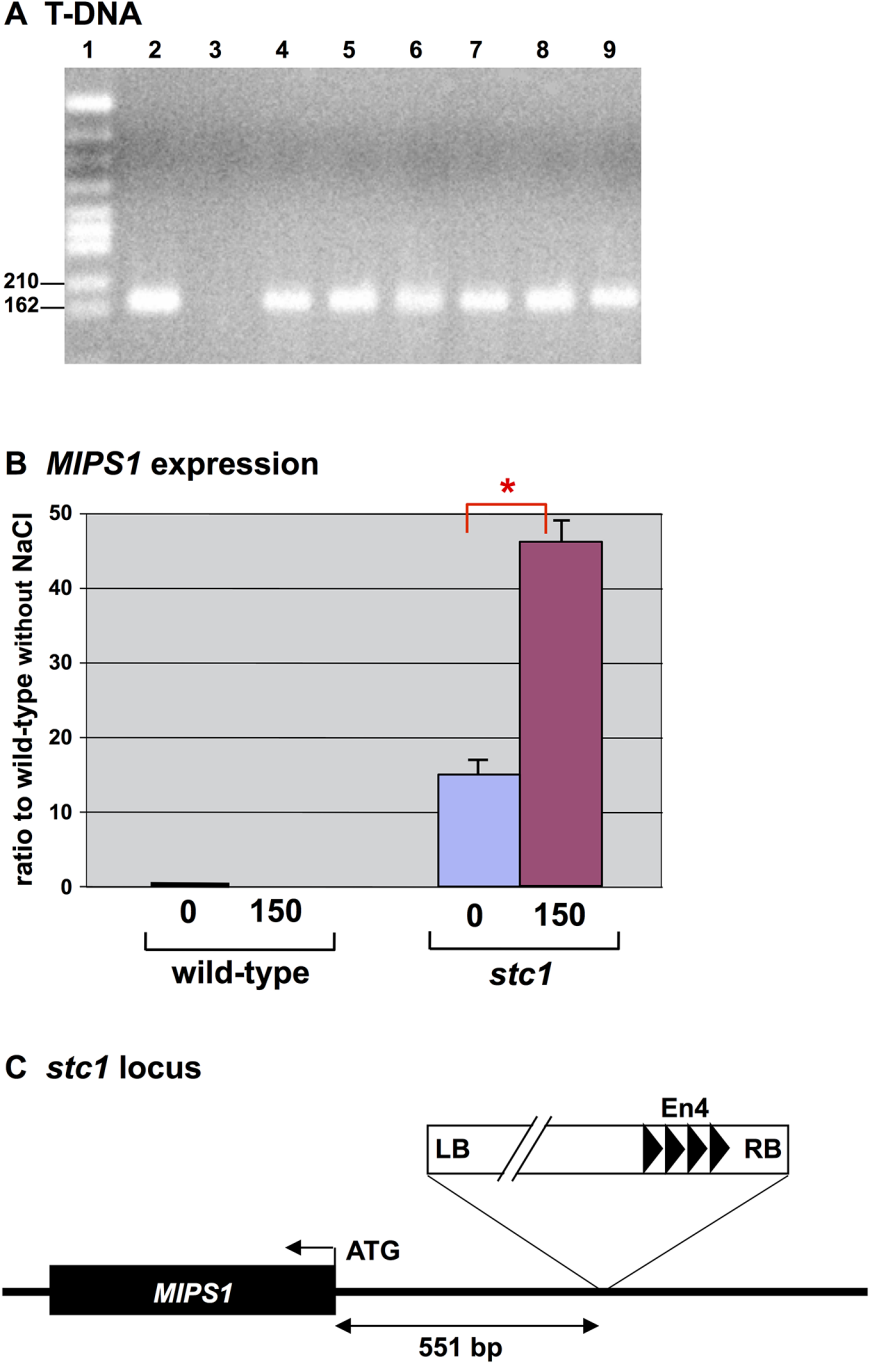
Confirmation of T-DNA integration in mutants. (A) The 200-bp genomic DNA product corresponding to P*35S*-*ALS-SU* ^r^ was detected in chlorsulfuron-resistant calli. Lane 1, M5 Marker (NipponGene); lane 2, positive control (vector pRi35ADEn4); lane 3, negative control (wild-type, *Arabidopsis* Col-0); lanes 4–9 *stc1*, *stc2*, *stc3*, *stc4*, *stc12* and *stc17*, respectively. (B) Transcript levels of *MIPS1* in calli. Total cellular RNA was extracted from 3-week-old calli grown on CIM without NaCl or with 150 mM NaCl. Transcript levels were determined by real-time PCR using the LightCycler (Roche) and normalized using the internal standard (*ACT2*). Error bars represent ± standard error (SEM) from three experimental replicates. Here is * for significant difference with *P* < 0.05. (C) A map around the insertion of *stc1* on chromosome 4.

T-DNA integration was confirmed by PCR and Southern blot analyses of chlorsulfuron-resistant calli. One of the mutant lines, *salt tolerant callus 1* (*stc1*), was intensively analyzed as an example for further molecular characterization. The PCR of DNA from *stc* mutants generated 200-bp fragment which were the same size as the product amplified from pRi35ADEn4 (Fig 2A), thereby confirming the transformation.

### Thermal Asymmetric Interlaced (TAIL)-PCR to Identify Activated Loci

**Fig 3:**
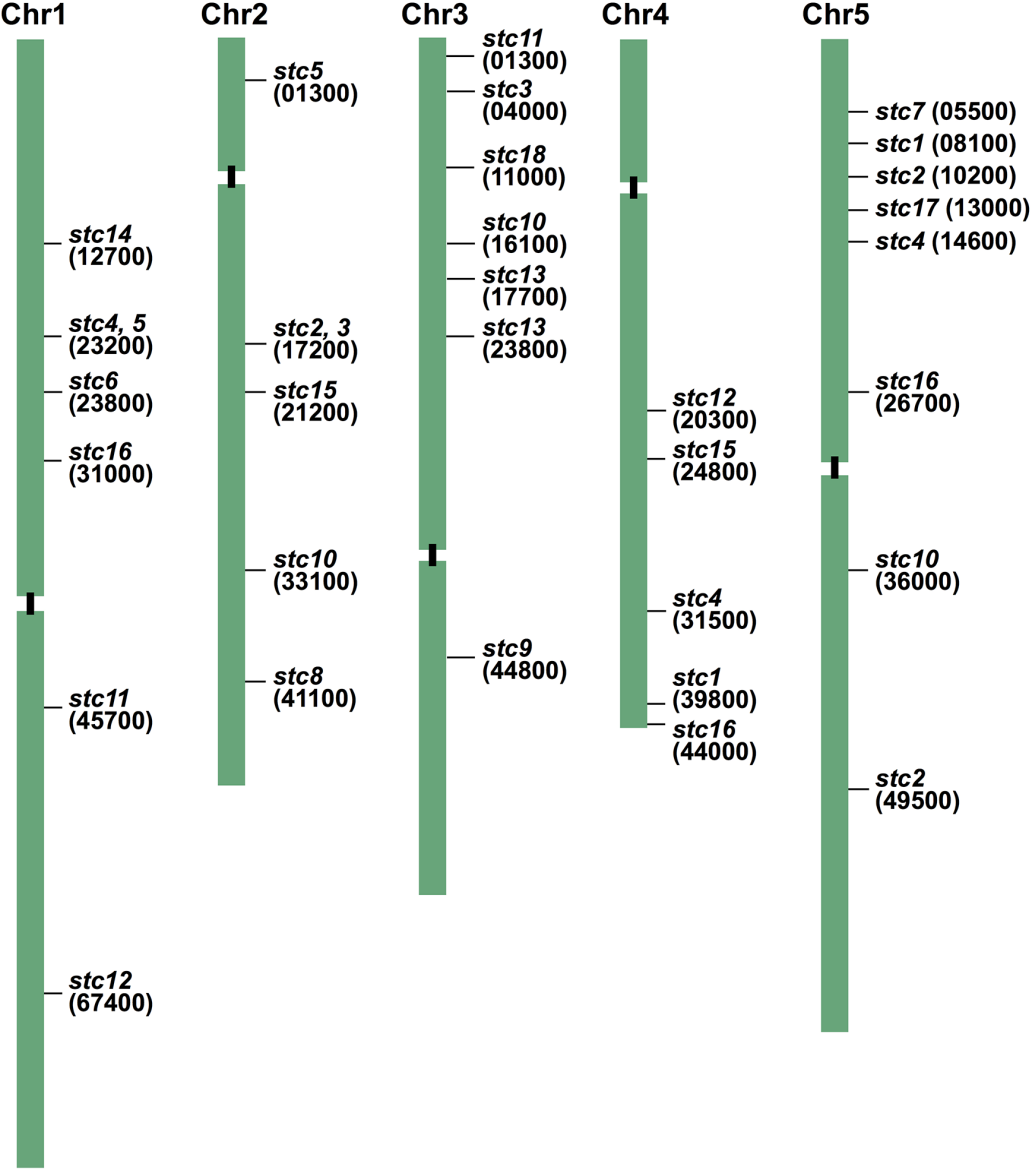
Representation of insertion sites on chromosomes in 18 mutants. The 5 digits in parentheses are those of AGI codes and correspond roughly to the positions where T-DNA was inserted, but not to genes exactly activated.

To determine the locations of T-DNA integration and activated genes, TAIL-PCR was performed for all *stc* mutants using primers from both ends of the T-DNA and arbitrary degenerate (AD) primers [15, 19]. The sequences obtained from the TAIL-PCR analyses were subjected to a BLAST search at NCBI, although we were unable to identify the insert locations by TAIL-PCR in some mutants. Specific primers were designed and used in combination with T-DNA-specific primers to confirm the insertion sites within the *Arabidopsis* genome. Screening of the mutants yielded a total of 33 insertions in 18 mutants (Fig 3). Two insertion sites were mapped in *stc1*. The T-DNA inserts in *stc1* were located on chromosomes 4 and 5. On the other hand, causal loci found in *stc2* and *stc3* were very close to At2g17200, and those of *stc4* and *stc5* to At1g23200 (Fig 3), suggesting that the activation tagging was nearly saturated. In *stc2* and *stc3*, there was no gene present between the two insertion points, whereas a region spanning a distance of 15 kbp was found between the two insertions in *stc5* and *stc4*. The structure of the T-DNA in three mutants was arranged in a complex manner and we were unable to map the insertions by TAIL-PCR. Additionally, in some mutants the right border of the T-DNA could not be mapped, which may have been due to the deletion of specific sequences in this region.

### Expression Profiles to Identify Activated Genes

The activation at 18 loci was verified through expression profiles. We examined the transcript level of genes adjacent to either end of the T-DNA (flanking 10-kbp regions to the left and right of the T-DNA) in calli that were stressed (150 mM NaCl) or growing on standard CIM in the absence of NaCl. The expression of *MIPS1* (At4g39800) encoding *myo*-inositol-1-P-synthase in chromosome 4 increased 45 or 15 times greater with or without 150 mM NaCl, respectively, in the *stc1* mutant in comparison with the wild-type (Fig 2B), and approx. 260 times higher in the mutant than the wild-type with salt which dramatically reduced the transcript in the wild-type. *MIPS1* was the closest gene at the left border of the T-DNA and was located a distance of 551 bps (Fig 2C). Any genes were not enhanced from the insertion present in chromosome 5, where the T-DNA was located within the open reading frame (ORF) of At5g08100.

An insertion next to At5g10170 (a homolog of *MIPS1*) was found in mutant *stc2*, although this gene was not enhanced. Additionally, genes located at both ends of the T-DNA in mutants *stc3*, *stc7* and *stc8* were activated, although the expression ratios differed and the distance between the activated genes and T-DNA ranged from 88 bp to 5,556 bp. On the other hand, genes that were located within 10 kbp from either end of the T-DNA in mutants *stc9*, *stc11* and *stc14* were not activated, although the activation of genes located beyond 10 kbp may occur. The ratio of activation of causally-related genes in the mutants ranged from 1.7 to 87.0 in the absence of NaCl, and from 0.8 to 250.0 in the presence of 150 mM NaCl. The gene involved in *stc8* has also been intensively investigated and its work is shown in the following article published together in PLOS ONE, doi: 10.1371/journal.pone.0126872.

### Salt Tolerance of Regenerated Plants and Their Calli

**Fig 4:**
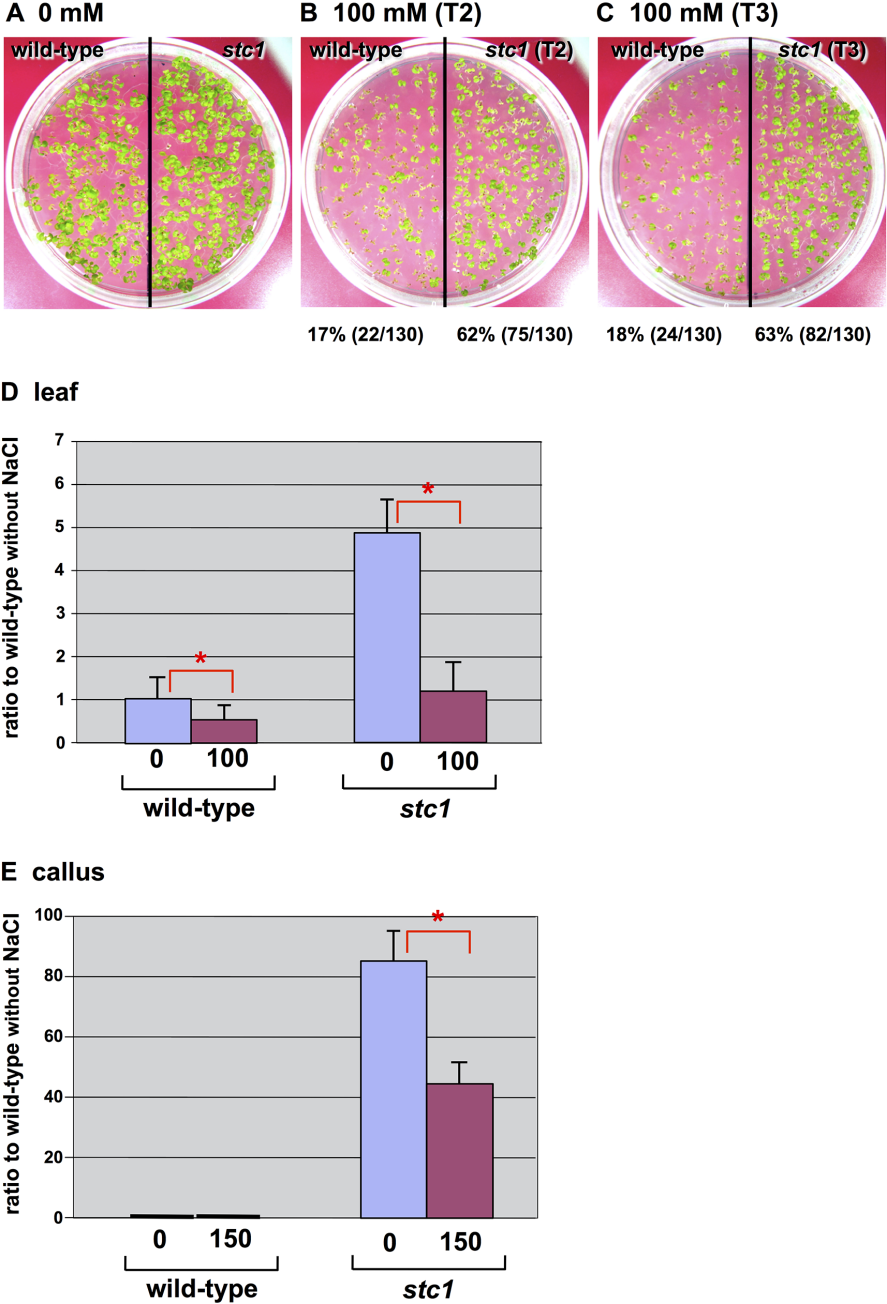
Phenotypes of wild-type and regenerated *stc1* plants on 100 mM NaCl medium. Seeds were surface-sterilized, kept at 4°C for 7 d and then transferred to MS supplemented with 100 mM NaCl. (A) *stc1* T2 seedlings on normal MS. (B) *stc1* T2 seedlings after 2 weeks at 100 mM NaCl. (C) *stc1* T3 seedlings after 2 weeks at 100 mM NaCl. Survival percentages are given in parentheses. (D) Transcript levels of *MIPS1* in the leaves of regenerated plants. Total cellular RNA was extracted from the leaves of plants with or without 100 mM NaCl stress. Transcript levels were normalized using the internal standard (*ACT2*) and are shown as ratios with respect to levels in the wild-type. Error bars represent ± standard error (SEM) from three experimental replicates. Here is * for significant difference with *P* < 0.05. (E) *MIPS1* expression in calli induced from roots of regenerated *stc1*. RNA was extracted from calli grown on CIM without NaCl or with 150 mM NaCl. Error bars represent ± standard error (SEM) from four experimental replicates. Here is * for significant difference with *P* < 0.05.

To further investigate whether genes responsible for salt tolerance at cellular levels also contribute to salt tolerance at differentiated plant levels, plants were regenerated from mutant calli and screened on different levels of salt. T2 and T3 seeds homozygous for activation tagging of *MIPS1* were screened on MS medium supplemented with 100 mM or 150 mM NaCl. Seedlings showed tolerance to NaCl based on criteria that included the number of surviving plants, the growth rate and root length, whereas wild-type plants were sensitive to NaCl (Fig 4A–4C). We generated calli from homozygous *stc1* plants and screened them on 150 mM NaCl. The expression of *MIPS1* in these calli was markedly higher both at standard and salt stress levels than in their leaves (Fig 4D and 4E).

### *MIPS1* as a Causal Gene for Salt Tolerance by Retransformation

**Fig 5:**
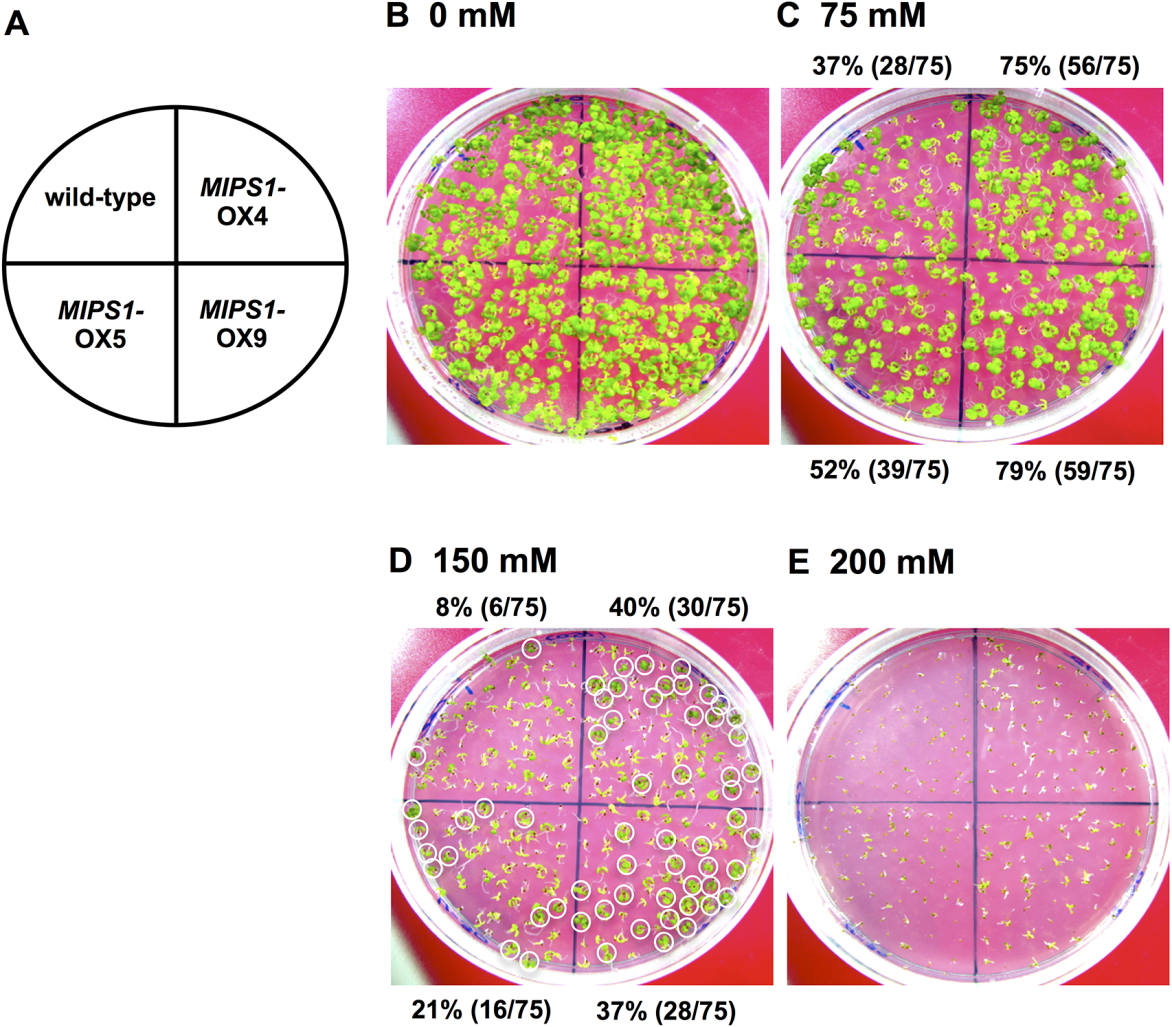
Phenotypes of wild-type and retransformed *MIPS1*-OX plants on medium containing different levels of NaCl. (B, C, D and E) Experimental plan, wild-type and three different homozygous lines for *MIPS1* on 0, 75, 150 and 200 mM NaCl, respectively. Survival percentages are given in parentheses. The surviving plants are circled with white lines in Panel D.

**Fig 6:**
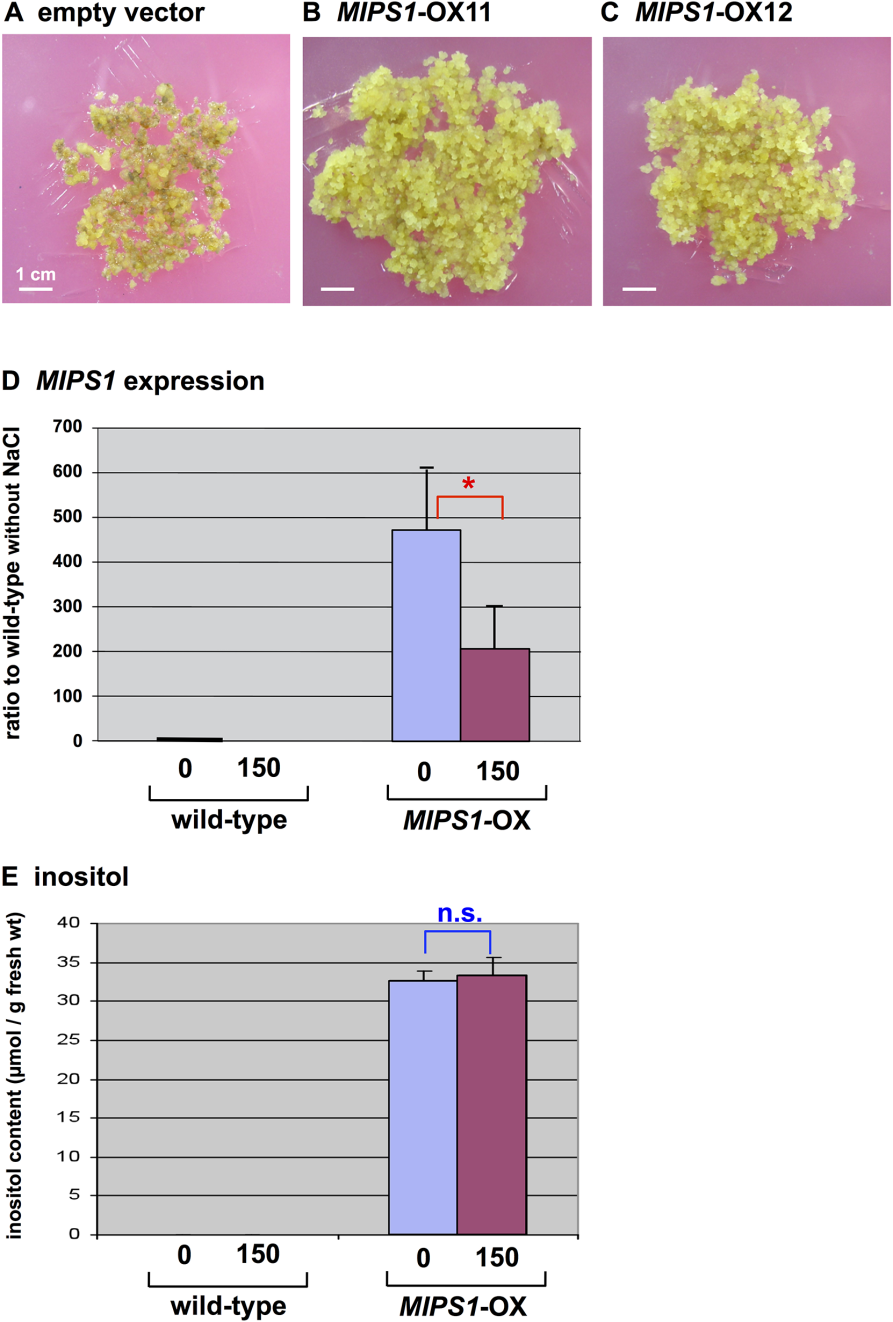
Phenotypes of calli of *MIPS1*-OX lines on medium containing NaCl. Lines transformed with an empty vector (A) and *MIPS1*-OX (B and C) were cultured on CIM with 150 mM NaCl after 3 weeks. (D) Transcript levels of *MIPS1* in calli of the wild-type and those directly transformed with *Agrobacterium* harboring pBCH1-EN-MIPS for *MIPS1*-OX. Total cellular RNA was extracted from 3-week-old calli grown on CIM with or without 150 mM NaCl. Error bars represent ± standard error (SEM) from four experimental replicates. Here is * for significant difference with *P* < 0.05. (E) Inositol content in calli of the wild-type and those directly transformed with the *MIPS1*-OX construct. In the wild-type we observed peaks a minute for inositol and their levels were as low as 0.9 ± 0.2 μmol / g fresh weight independently of salt concentrations. Error bars represent ± standard error (SEM) from three experimental replicates. Here is “n.s.” for no significant difference.

We have transformed wild-type *Arabidopsis* with a construct to over-express *MIPS1* under the control of the CaMV 35S promoter. Plants were transformed either by a floral-dip method or through the roots, and resultant transgenic plants or calli were subjected to analyses. The germination of transgenic seeds was higher than that of the wild-type on MS plates supplemented with 75, 100, 150 and 200 mM NaCl. Wild-type plants exhibited a considerably reduced number of growing seedlings at 150 mM NaCl (Fig 5D). Among the transformed over-expression (OX) lines, OX5 line was less tolerant than OX4 and OX9 lines at 75 mM and 150 mM NaCl (Fig 5C and 5D). At 200 mM NaCl, very few wild-type seeds were able to germinate, whereas most seeds of OX lines germinated after 1 week (see dead seedlings, Fig 5E). Calli induced from OX lines homozygous for transgenic *MIPS1* were grown for 7 weeks on CIM containing 150 mM NaCl. The calli of OX lines grew more vigorously than the wild-type under the salt stress (Fig 6A–6C). The overall results clearly indicate that *MIPS1* is responsible for salt tolerance in plants and calli.

### Expression and Function of Transgenic *MIPS1* in Plants and Calli

**Fig 7:**
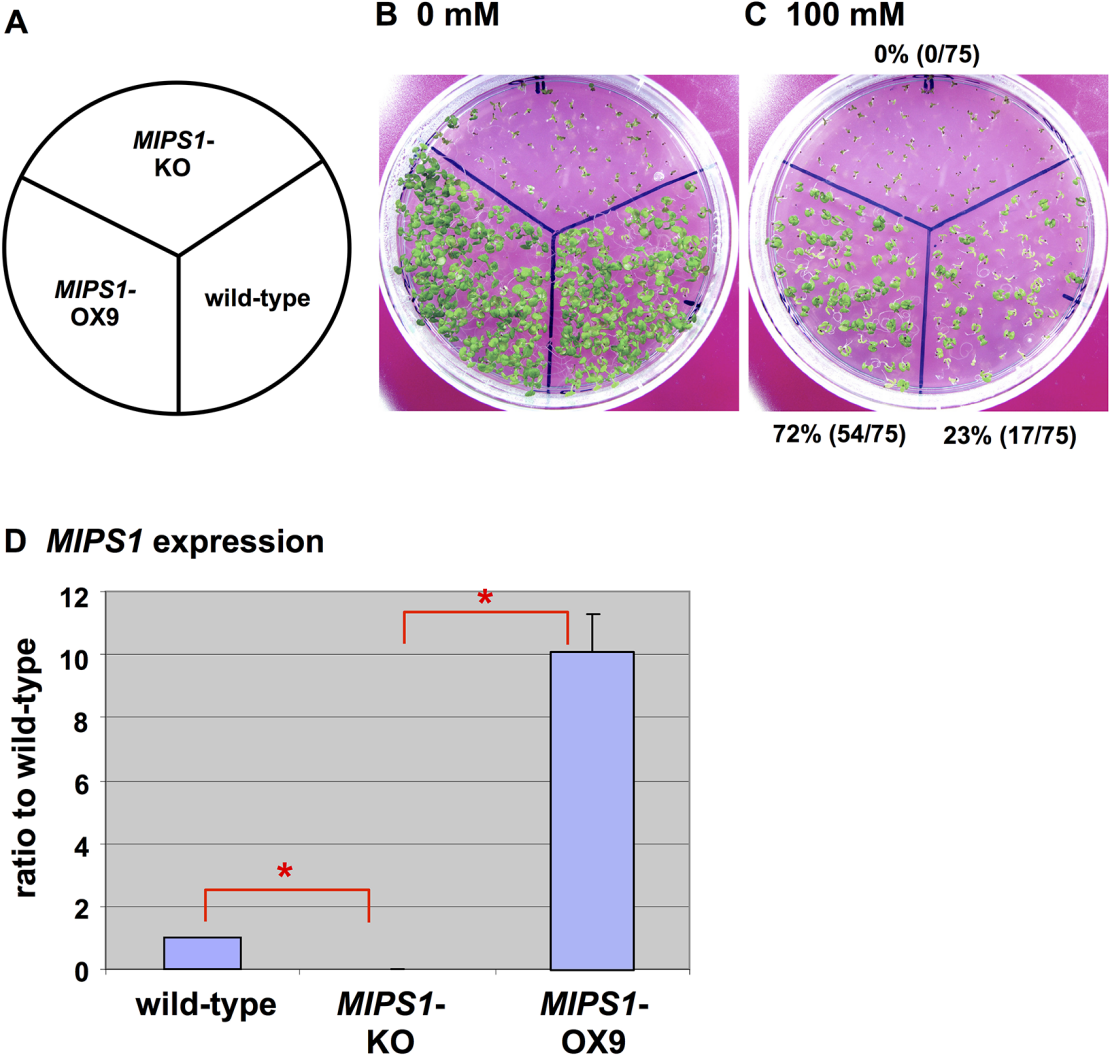
Phenotypes of wild-type, *MIPS1*-KO and retransformed MIPS1-OX lines with different levels of NaCl. (B and C) Plants were grown in the absence (B) or presence of 100 mM NaCl (C), respectively, for 3 weeks. Survival percentages are given in parentheses. (D) Transcript levels of *MIPS1* in the leaves of wild-type, KO and OX lines without salt stress. Transcript levels were determined by real-time PCR using the LightCycler (Roche) and normalized by the internal standard (ACT2). Error bars represent ± standard error (SEM) from three experimental replicates. Here is * for significant difference with *P* < 0.05.

RT-PCR was performed to analyze the expression of *MIPS1* in *MIPS1*-OX homozygous plants and calli. RNA was extracted from plants grown without salt stress. The transcript level was 10 times higher in the transgenic line, OX9, than in the wild-type (Fig 7D). Additionally, RNA was prepared from calli incubated with or without 150 mM NaCl. The expression of *MIPS1* was almost 480-fold higher in the retransformed OX calli compared to the wild-type in the absence of NaCl (Fig 6D), and were approx. 1,800 times higher in the OX calli than the wild-type with salt which dramatically reduced the transcript in the wild-type. Therefore, the expression found in differentiated plants was markedly lower compared with that measured for calli.

The total inositol was extracted from calli and subjected to gas chromatography after hydrolysis with H_2_SO_4_. The content of total inositol was approx. 30 μmol/g fresh weight in transgenic calli regardless of salt stress conditions, while in the wild-type we observed peaks a minute for inositol as low as 0.9 ± 0.2 μmol / g fresh weight independently of salt concentrations (Fig 6E).

### Phenotypes of the *MIPS1*-Knockout (KO) Line

To further analyze the function of MIPS1, we examined the *MIPS1*-KO line, SALK_023813, which is only an available line. After a few plants of this line homozygous for the KO locus grew on MS containing 2% sucrose, they were transferred to soil so that seeds could be collected. Most individuals of this line exhibited impaired germination or development of seedlings and roots on MS medium even with sugar (Fig 7B and 7C). Although there are two additional homologs of *MIPS1* (At4g39800) in *Arabidopsis*, At2g22240 and At5g10170, sharing 93.5% and 89.4% sequence identity to *MIPS1*, respectively. The homozygous *MIPS1*-KO line showed an undetectable level of *MIPS1* expression in plants (Fig 7D). In contrast to the KO plants, the *MIPS1*-OX line displayed greater tolerance than the wild-type at 100 mM NaCl (Fig 7C).

**Fig 8:**
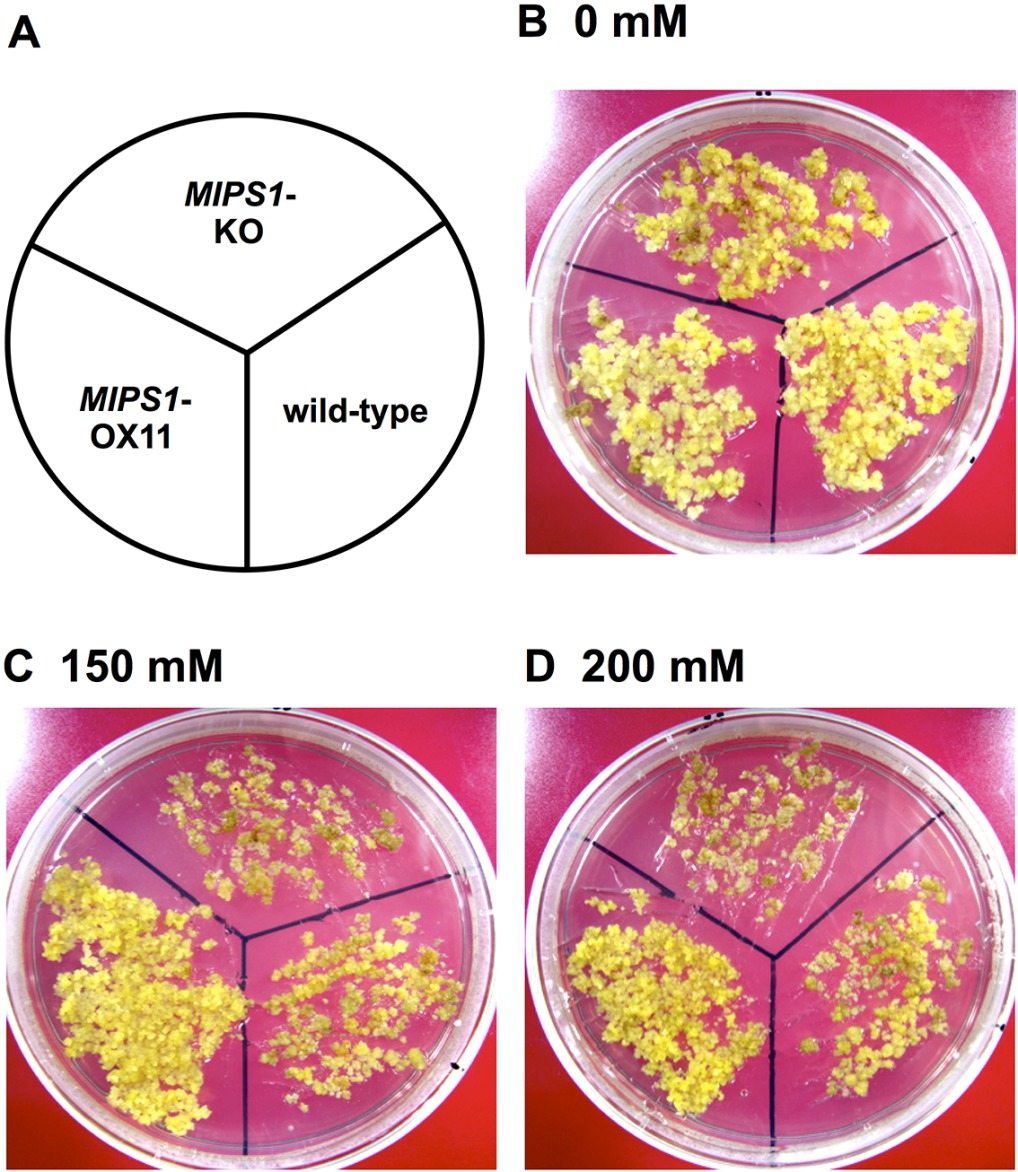
Phenotypes of calli of wild-type (WT), *MIPS1*-KO and retransformed MIPS1-OX lines with different levels of NaCl. (B, C and D) Calli cultured on medium containing 0, 150 and 200 mM NaCl, respectively.

From the very short roots of germinating seedlings of the KO plants, we generated calli and subsequently grew them on 125, 150 and 200 mM NaCl (Fig 8). The growth of calli was unabated in the absence of NaCl stress (Fig 8B), whereas the growth of KO calli was severely affected at 150 mM NaCl. Growth of wild-type calli was also disturbed at 150 mM, but not as severely as that of the KO line, while the growth of the OX line was almost unaffected (Fig 8C). The growth of the OX line was less affected at 150 mM or 200 mM NaCl, whereas calli of the wild-type were retarded in growth and all KO calli had died (Fig 8D). Calli of the OX line showed more tolerance than differentiated plants.

### MIPS1 Orthologs

**Fig 9:**
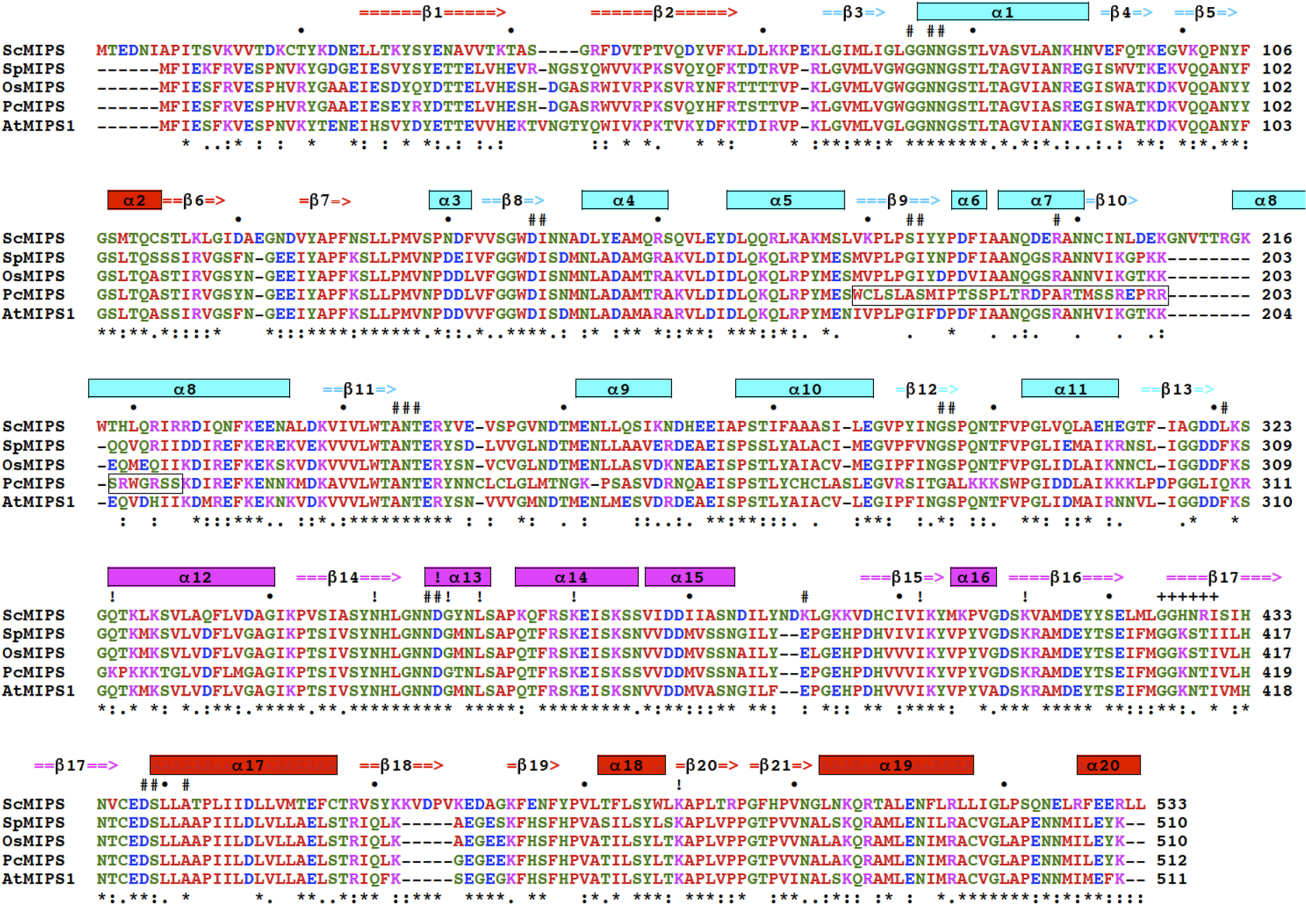
The alignment of amino acid sequences of AtMIPS1 and others. The following MIPSs are shown: ScMIPS, *Saccharomyces cerevisiae*; SpMIPS, *Spirodela polyrrhiza*; OsMIPS, *Oryza sativa*; and PcMIPS, *Porteresia coarctata*. The completely conserved residues are indicated with “*” and those that are essentially conserved are shown with “**. *“*** or “**: “**, and the amino acids are colored red, hydrophobic; green, hydrophilic; purple, positively charged; and blue, negatively charged. Elements of secondary structure with reference to the crystal structure of ScMIPS [61] are shown as cylinders for helices, and arrows for b-strands. These elements are also colored domain by domain: red, the central domain (Met1-Val60, Gly104-Val133 and Glu439-Lys511); cyan, the NAD-binding domain (Pro61-Phe103 and Asn134-Ser310); and magenta, the catalytic domain (Gly311-Asp423). Amino acids that interacted with NAD in ScMIPS are indicated with “# “and those that interacted with 2-deoxy-glucitol-6-phosphate (analog of glucose-6-phosphate, an inhibitor of MIPS) are shown with “! “. A putative myristoylation site conserved in PcMIPS and AtMIPS1 is marked with “+ “. The region of PcMIPS (Trp174-Ser210) reportedly involved in salt tolerance [43] is enclosed with black rectangles. Accession numbers of sequences: ScMIPS, 1JKI (Protein Data Bank); SpMIPS, Z11693; OsMIPS, AB012107; PcMIPS, SAF412340; and AtMIPS1, NM_120143.

MIPS amino acid sequences from different plant species are aligned in Fig 9. Two different MIPS genes have recently been reported in rice, the salt-tolerant *PcINO1* (*PcMIPS* from *Porteresia coarctata*) and the salt-sensitive *OsINO1* (*OsMIPS* from *Oryza sativa*). A region spanning 37 amino acids is thought to be responsible for the observed salt tolerance imparted by PcMIPS, as determined by deletion and insertion experiments [26].

## Discussion

### Salt Tolerance in Dedifferentiated Cells

Although detailed knowledge concerning the complex network of salt stress-signaling pathways in plants at the molecular and cellular levels remains to be determined [27], continued investigations and the development of *Arabidopsis* genomic tools have assisted in this task. Most investigations of salt tolerance have been conducted with differentiated plants. For example, we have screened a *photoautotrophic salt tolerance 1* (*pst1*) mutant, in which the activities of superoxide dismutase and ascorbate peroxidase were enhanced and led to the detoxification of reactive oxygen species (ROS) generated during photosynthesis in leaves [9]. This focuses attention on tissue-specific salt tolerance, which has recently been discussed in terms of SOS pathways mediated by SOS3 in roots and SCABP8 in shoots [11].

The mechanisms underlying salt tolerance have been investigated using a variety of experimental approaches: unicellular organisms such as *Escherichia coli* and *Saccharomyces cerevisiae* have been employed as model systems, comprising salt regulatory components at cellular levels and investigated with a view to utilizing information gleaned from these studies for the investigation of plants [28]. However, the comparison between these unicellular organisms and differentiated multicellular ones such as plants is limited because unicellular organisms constitute a more primitive intracellular compartmentation with a lack of some post-translational protein modifications and the capacity for cell-to-cell interactions. Therefore, we focused on dedifferentiated cultured cells of plants to investigate salt tolerance. While a detailed analysis of genes responsible for salt tolerance is underway and involves around 18 loci identified in this investigation, genes well characterized for salt tolerance do not exist around those loci, suggesting that novel genes for salt tolerance would be associated with the found loci. We have analyzed MIPS1 intensively, showing that it confers salt tolerance on differentiated plants and calli. Therefore, the strategy developed in this work may prove to be invaluable for the detection of new genes for salt tolerance.

### Efficiency with Activation Tagging

The employment of dedifferentiated calli for the selection of mutants has another advantage. That is, it is more efficient in regard to saving time and space for plant growth to prepare a set of tagged lines directly as calli without regeneration of plants, than to maintain seeds harvested from differentiated plants. This strategy has worked efficiently and resulted in the screening of a large number of mutants (ca. 62,000 during a period of 6 to 7 months) and the identification of ca. 40 lines from our initial selection, and finally 18 lines following a secondary screening. After mapping the insertions in all 18 lines using TAIL-PCR, we found one insertion site (At1g23160) common in *stc4* and *stc5*, and another insertion site (At2g17240) common in *stc2* and *stc3* (Fig 3), indicating that activation tagging was nearly saturated or that T-DNA insertion-site preferences are present in the genome. Additionally, our procedure has the advantage of uniform and reproducible conditions of stress and the selection of all mutants. Our strategy is also applicable for other species such as trees, where it is difficult to evaluate salt tolerance at whole-plant levels.

It is notable that there were some cases in which the enhancer sequences harbored in the T-DNA did not work. We observed that calli of *stc1* and *MIPS1*-retransformed OX lines were tolerant to 150 mM NaCl and marginally tolerant to 200 mM NaCl. The *stc1* mutant possessed two insertions, one on chromosome 4 and another on chromosome 5. The insertion on chromosome 4 resulted in enhanced expression of *MIPS1*, whereas no genes were enhanced as a result of the insertion on chromosome 5, perhaps as a result of methylation of the T-DNA [29] and subsequent transcriptional silencing. A similar result was observed in other mutants harboring two or three copies of T-DNA and where no gene was found to be enhanced. The gene silencing-impaired or single copy T-DNA lines have been recommended for activation tagging.

### *MIPS1*-KO Line

To glean insight into the role of MIPS1, we obtained a homozygous *MIPS1*-KO line. Most seeds of the KO plants were not viable (Fig 7). We assume that the change in seed viability was probably due to reduced levels of phytic acid, which is responsible for the storage of phosphates in seeds. Phytic acid, phytin or phytate, the most abundant form of phosphate in seeds, is synthesized through the sequential phosphorylation of inositol phosphates [30–34]. The metabolic pathway leading to the synthesis of phytic acid is unique to plants [35]. Although phytic acid is required for seed development, reduced phytate levels in maize [33], barley [36], rice [37] and soybean [38] did not affect seed development. Additionally, reduced phytic acid, increased *myo*-inositol and diminished *myo*-inositol phosphate intermediate levels in seeds had very little effect on plant growth and development [39]. However, phytase activity has been observed during seed maturation and development in barley, and in soybean a steady increase in phytic acid levels was observed until late seed maturation [40, 41]. Moreover, it has recently been shown that the silencing of *MIPS1* expression using RNAi impaired seed development and reduced phytic acid levels in transgenic soybean. Therefore, we conclude that reduced *MIPS1* expression interferes with seed development in the *Arabidopsis* KO line.

### Transcripts for *MIPS1* and their Function

Transcripts for *MIPS1* seem to be induced by salt in original activation-tagged *stc1* calli (Fig 2B), whereas they were rather suppressed in leaves and calli of third generation of *stc1* (Fig 4D and 4E) and in calli directly transformed with pBCH1-EN-MIPS for *MIPS1*-OX followed by continuous culture (Fig 6D). We suppose that the simulative induction of *MIPS1* gene expression would occur in the original calli by infection with *Agrobacterium tumefaciens* harboring construct for the activation-tagging. This speculation was supported by results published by Lee et al. [42] and the database ArrayExpress (http://www.ebi.ac.uk/arrayexpress/experiments/E-NASC-21/), where *MIPS1* was induced 1,200 times at its maximum until 35 days after infection with *Agrobacterium*. *MISP1* is also salt inducible up to a double level as known on the basis of the database eFP (http://bar.utoronto.ca/efp/cgi-bin/efpWeb.cgi). Synergistic enhancement of *MIPS1* transcripts with *Agrobacterium* infection in the presence of salt is supposed. On the other hand, a reduction of the transcript levels when plants and calli of third generation of *stc1* mutant and *MIPS1*-OX calli were exposed to salt (Figs 4D, 4E, and 6D), could be explained as no direct effect of *Agrobacterium* infection and rather harmful effect of NaCl to plant growth, resulting in lower accumulations of the transcripts.

The level of transcripts for *MIPS1* in calli transformed with the *MIPS1*-OX construct was approx. 480 or 210 times higher without or with NaCl, respectively, than that of wild-type without the salt stress (Fig 6D), while levels of inositol were nearly equal independently of salt concentrations (Fig 6E). We understand that *MIPS1* expression in the *MIPS1*-OX calli without salt made the inositol content almost saturated up to the highest level. We suppose that a limiting step for inositol levels would be the capacity of substrates for inositol biosynthesis. The reaching level of inositol is thought to be necessary for salt tolerance in the presence of salt.

### Involvement of MIPS1 in Salt Tolerance

The role played by polyols and sugar alcohols such as pinitol, mannitol, ononitol and methyl inositol as osmoprotectants against salt stress in various plants is well established [26, 43–46]. In higher plants, the inositol that accumulates in response to salt stress may function as an osmolyte in addition to its critical roles concerning the cellular machinery [5]. The extent to which osmolyte levels rise is dependent on changes in the external osmotic potential [3, 47]. Osmolytes are believed to possess two major functions that include osmotic adjustment at high concentrations and unknown roles at low levels. In the former, osmolytes are believed to maintain an osmotic balance but are also thought to contribute towards the stabilization of proteins and cell structures, thus maintaining growth at high salinity [48]. Whether osmolytes play a role in scavenging ROS remains unclear [2, 47, 49–52].

Compounds such as gums, cell wall-located carbohydrates, glycoproteins and mucilages, which are involved in protective functions during stress, are also produced from inositol and inositol-1-phosphate. Mannitol, a straight-chain polyol, is reportedly involved in the scavenging of hydroxyl radicals and stabilization of macromolecular structures through hydrogen bonding [51–54]. Moreover, the accumulation and role of *myo*-inositol and its derivatives under abiotic stress have also been discussed [5, 30, 46, 55]. Although it has been shown that accumulation imparts osmoprotection, the accumulation of inositol and its derivatives under salt stress has not been observed in *Arabidopsis* [44], and it may be that these species are employed in the abiotic stress-induced synthesis of galactinol and raffinose [56].

MIPS1 network genes have recently been summarized on the basis of previous microarray studies for different biotic and abiotic stresses. Integration of various genome-wide transcript profiles for the stresses through the fuzzy k-means clustering method [57] has revealed that a large number of signaling molecules emerge from membrane phospholipids, and their roles in osmotic stress responses have been well established. A number of genes such as those for inositol polyphosphate 5-phosphatase II, FYVE domain-containing phosphatidylinositol-4-phosphate 5-kinase (PI4P5K) and lipase class 3 family protein are induced. This finding also suggests the importance of inositol or its derivatives in the multiple abiotic stresses. In another effort to establish the gene networks based on the graphical Gaussian model (GGM) for biochemical and stress responses [58], an array of genes was found to be associated with *MIPS1*, many of which may be crucial players for stress tolerance like Δ-1-pyrroline-5-carboxylate synthetase A (*P5CS1*), heat shock protein 100 (*Hsp100*), HSP 70-like protein and CBL-interacting protein 7 (*CIPK7*).

### Variation of Salt Tolerance by MIPS1 Orthologs

Introduction of *PcMIPS* into *E*. *coli*, *Schizosaccharomyces pombe*, rice, tobacco and brassica confers salt tolerance; however, *OsMIPS*-transformed tobacco plants were not tolerant compared with *PcINO1*-transgenic plants [26]. Similarly, over-expression of inositol-3-phosphate synthase from *Spirodela polyrrhiza* (SpMIPS), which resulted in up to a 4-fold increase in free inositol levels, was less associated with an increasing salt tolerance in *Arabidopsis* [23], although it was argued that expression was not high enough to show significant results. In addition to these reports concerning enhancement of salt tolerance resulting from the over-expression of MIPS homologs, similar effects were observed in transgenic tobacco and *Arabidopsis* plants following the overproduction of mannitol [59, 60]. We over-expressed *MIPS1* under the CaMV 35S promoter with 4 copies of its enhancer and observed an increase in salt tolerance in *Arabidopsis* compared with the wild-type.

The *PcMIPS* reported by Majee et al. [26] and *AtMIPS1* as revealed in this investigation conferred salt tolerance on plants, whereas *OsMIPS* [26] and *SpMIPS* [23] did not. When we compared the protein sequence of AtMIPS1 with those of PcMIPS, OsMIPS and SpMIPS, we found that AtMIPS1 possessed amino acid sequences highly identical to those of SpMIPS and OsMIPS, although it was less homologous especially in the region consisting of Trp174-Ser210 in PcMIPS (Fig 9), which reportedly is responsible for salt tolerance [43]. This region contributes to the creation of exteriors of the NAD-binding domain, and is common to SpMIPS, OsMIPS and AtMIPS1 in their electrostatic surface potential but not to PcMIPS. In our search for identical local structures in AtMIPS1 and PcMIPS which make plants salt tolerant, a putative myristoylation site composed of GGKNTI at 410–415 in AtMIPS1 was found, leading to the speculation that it may play a role in the regulation of intercellular localization of MIPS or its interaction with some specific protein(s).

### Function of MIPS1

We found that higher expression of *MIPS1* also confers salt tolerance on calli and plants of *Arabidopsis*. The tolerance depends upon the expression level because calli that showed the highest expression exhibited much more tolerance than plants in which the expression was low (Figs 5, 6, 7 and 8). Over-expression of *MIPS1* was also found in plants regenerated from *stc1* calli and which were tolerant to 75, 100 and 150 mM NaCl. Therefore, we conclude that induction of *MIPS1* expression leads to salt tolerance in calli and plants. Activation of *MIPS1* using the CaMV 35S enhancers resulted in the induction of *MIPS1* expression by up to 15-fold relative to that on standard medium (no NaCl), and a 45-fold increase in *MIPS1* expression at 150 mM NaCl in *stc1* compared with the wild-type (Fig 2B). One possible interpretation of these results is that activation of *MIPS1* expression leads to an increased shunting of glucose-6-phosphate into *myo*-inositol biosynthetic pathways, which ultimately results in the accumulation of free inositol. When we analyzed the inositol content in calli in the *MIPS1*-OX line and the wild-type with or without stress, the inositol content was much higher in *MIPS1*-transgenic calli compared with the wild-type. These higher levels of inositol resulted in salt tolerance both at calli and plant levels. However, as in plants, the expression of *MIPS1* was significantly reduced compared with calli, and the tolerance level therefore was also reduced in plants. An increase in inositol, d-ononitol and d-pinitol, the upregulation of *MIPS1* mRNA levels, and an increase in free inositol have been reported for a variety of plants including the halophyte common ice plant and other transgenic plants [8, 44–46]. On the other hand, no upregulation of *MIPS1* was observed in *Arabidopsis* plants during salt stress [44], although upregulation of *MIPS1* was observed in salt stress [8] where upregulation of the same gene (At4g39800) was observed under 250 mM NaCl stress for 2 h. Therefore, we conclude that the higher expression of *MIPS1* to promote the inositol content, which is nearly 10-times higher than that of *Arabidopsis* transgenic with *SpMIPS* [23], protects calli and plants from salt stress as osmosolutes or through the supply of a precursor for regulation in signal transduction pathways.
